# A systematic review of the role of school-based healthcare in adolescent sexual, reproductive, and mental health

**DOI:** 10.1186/2046-4053-1-49

**Published:** 2012-10-26

**Authors:** Amanda J Mason-Jones, Carolyn Crisp, Mariette Momberg, Joy Koech, Petra De Koker, Cathy Mathews

**Affiliations:** 1 Health Systems Research Unit, South African Medical Research Council, Cape Town, South Africa; 2 Global Public Health, Department of Health Sciences, The University of York, Seebohm Rowntree Building, Heslington, YO10 5DD, UK; 3 Adolescent Health Research Unit, University of Cape Town, Cape Town, Western Cape, South Africa; 4 Adolescent Health Research Unit, University of Cape Town, Cape Town, South Africa; 5 Brown University, Providence, USA; 6 International Centre for Reproductive Health, Ghent University, Ghent, Belgium

**Keywords:** School-based health care, School-based health clinics, Adolescent, Adolescent health services, School-based health centers, Sexual and reproductive health, Mental health, Systematic review

## Abstract

**Background:**

Accessible sexual, reproductive, and mental healthcare services are crucial for adolescent health and wellbeing. It has been reported that school-based healthcare (SBHC) has the potential to improve the availability of services particularly for young people who are normally underserved. Locating health services in schools has the potential to reduce transport costs, increase accessibility and provide links between schools and communities.

**Methods:**

A systematic review of the literature was undertaken. Pubmed, Psychinfo, Psychnet, Cochrane CENTRAL, and Web of Science were searched for English language papers published between January 1990 and March 2012

**Results:**

Twenty-seven studies were found which fitted the criteria, of which, all but one were from North America. Only three measured adolescent sexual, reproductive, or mental health outcomes related to SBHC and none of the studies were randomized controlled trials. The remaining studies explored accessibility of services and clinic utilization or described pertinent contextual factors.

**Conclusions:**

There is a paucity of high quality research which evaluates SBHC and its effects on adolescent sexual, reproductive, and mental health. However, there is evidence that SBHC is popular with young people, and provides important mental and reproductive health services. Services also appear to have cost benefits in terms of adolescent health and society as a whole by reducing health disparities and attendance at secondary care facilities. However, clearer definitions of what constitutes SBHC and more high quality research is urgently needed.

## Background

Access to healthcare, especially for adolescents, is a high priority policy objective in many countries and particularly for sexual and reproductive health [1] and mental healthcare [2]. In 2009 young people aged 15 to 19 years accounted for 41% of all new HIV infections globally and more than half of other sexually transmitted infections (STIs) [3]. It has also been estimated worldwide that 11% of those who give birth each year are adolescents [4]. Mental health problems are estimated to affect 10% to 25% of adolescents globally, yet their mental healthcare is often neglected [5,6]. Clearly sexual and reproductive health services, and mental healthcare services need to be easily accessible to adolescents, and the barriers to access [7] overcome.

At the turn of the 20th century social activists in the US led the movement to serve the needs of young people living in disadvantaged communities by providing health and social services through schools, though service was often through voluntary efforts and rarely formally incorporated into wider health systems [8]. The impulse to provide such services emerged over the years from the realization that young people’s health status and their educational achievement are closely related [9] and from the need to provide an accessible consumer-oriented service [10]. The advantages of schools as a location for delivering healthcare services are clear: schools are where most young people are, they are accessible to families, can provide a comprehensive and non-stigmatizing health service and can provide links between schools and communities.

In recent years, formal health services have been developed in the school setting such that a ‘one-stop shop’ delivers a comprehensive, integrated preventative health service providing medical, nursing, and mental healthcare to young people [10,11]. This model of healthcare delivery has gained popularity in the United States (US) particularly. The National Assembly on School-Based Health Care (http://www.nasbhc.org) found almost 2,000 school-based healthcare (SBHC) services being implemented country-wide in 2008. Nevertheless, despite their popularity in the US, the provision of services remains patchy with care provided to approximately 2% of young people enrolled in schools [12]. Evidence suggests that SBHC is also common in the UK, involving nursing services only, but services are unevenly distributed and outcomes rarely documented [13]. With regard to other countries, particularly middle and low income countries, there is virtually no documented information. South Africa, is currently embarking on the development of SBHC as part of its primary healthcare re-engineering program [14].

Known variously as ‘school-based health care (SBHC)’, ‘school-based health clinics’, or ‘school-based health centers’ (SBHCs), this way of delivering healthcare is considered to be one of the most effective strategies for delivering comprehensive primary and preventative health services to young people, especially those that are normally underserved by health services [15-17]. Although they would not normally reach those young people who have already dropped out of school, are homeless, or incarcerated [18] there is evidence that they can prevent school-dropout and the development of risky behaviors [19,20].

SBHC aims to provide essential primary care services, overcome barriers such as transport issues, limited community services, and inconvenient location or appointment systems, and can also act on the multiple determinants of health, including public health interventions and environmental change strategies [21], Provision can vary from fully equipped and permanently staffed centers with medical, nursing, and auxiliary staff [22] to clinics offering nursing services for only a few hours per week [23].

Although literature exists on school health services as a whole [13,24-26] which include comprehensive services based at schools, dedicated adolescent health services, school-linked services based at local health centers and servicing a number of schools and other outreach services there is, to our knowledge, no known existing review of the role of school-based healthcare, that is, located on the school grounds and serving the students therein. Specifically, we wanted to review the evidence of the effects and cost-effectiveness of SBHC on adolescent sexual and reproductive health and mental health; issues key at this age. We wanted to look at effectiveness of SBHC, to review factors influencing young people’s use of SBHC, and to describe pertinent contextual facilitating and impeding factors in the establishment of SBHC.

## Methods

Pubmed, Psychinfo, Psychnet, Cochrane CENTRAL, and Web of Science were searched for English language papers published between January 1990 and March 2012 using the search terms ‘School-based health care’, ‘School-based health service’ and ‘School-based health clinic’, ‘school-based health centre’, with the addition of the subterms: adolescents, youth, high schools, health, prevention, HIV, mental health, reproductive health, and sexual health. We chose not to review studies from before 1990 as we wanted to review models of SBHC which were relevant to current health system provision.

Search strategy:

Databases: Pubmed, Psychinfo, Psychnet, Cochrane CENTRAL, Web of Science

Inclusion dates: 1 Jan 1990-31 March 2012

Language: English

Search terms:

#1“Adolescent/s”

OR

#2“Youth”

AND

#3 “School based health services”

OR

#4 “School based health care”

OR

#5“School based health centre”

OR

#6“School-based health clinic”

OR

#7 “Health services” AND “High schools” AND “Prevention” AND “HIV”

AND

#8 “Mental health”

OR

#9 “Reproductive Health”

OR

#10 “Sexual health”

OR

#11“Sexual and Reproductive Health”

Studies selected included any process and/or outcome studies using quantitative or qualitative methods that described evaluations of SBHC involving adolescents in secondary schools/high schools. Studies included evaluations of school-based health centers, school-based health clinics, or school-based healthcare. Evaluations of so-called ‘school-linked’ service evaluations were excluded if the school was not the primary location of the service. Primary outcomes included sexual and reproductive health and mental health outcomes and secondary outcomes included satisfaction with services, accessibility of services, measures of use, facilitating and impeding factors, and cost-related analyses. Titles of all possible papers and the abstracts were reviewed. Full papers of identified titles were screened by at least two of the reviewers to determine if they met the inclusion criteria (AMJ and either CM/MM/JK/PDK or CC). Data were extracted from reports that met the inclusion criteria using a piloted form and this was done by two of the review authors (AMJ and either CC or MM) independently.

## Results

**Figure 1:**
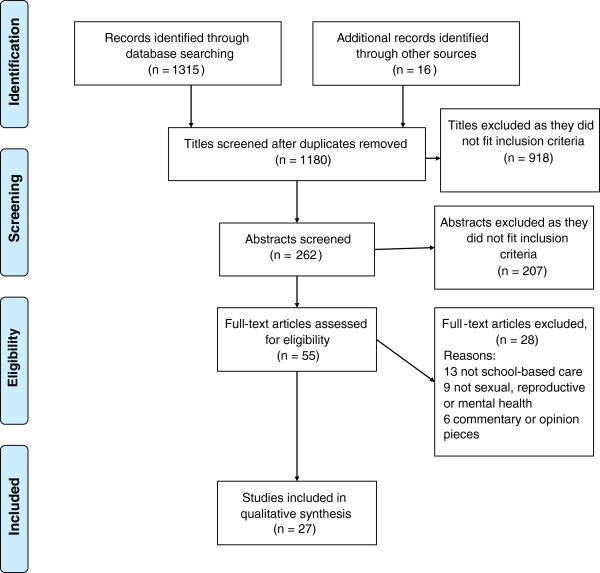
Study flow diagram.

A total of 1,331 titles were identified through searching databases (*n*=1,315) and through other sources (*n*=16) of which 151 duplicates were removed. All titles were screened and 918 articles were excluded as they did not meet the inclusion criteria. Of these, 262 articles were identified and abstracts were screened as being potentially eligible for inclusion. However, at this stage 207 did not meet the inclusion criteria and 55 full text articles were assessed for eligibility. Of these, 28 articles were eventually excluded. In total, 27 studies were included in the final review (see Figure 1).

Characteristics of included studies and evaluation findings are shown in Table 1. Apart from one study that was conducted in the UK, all were conducted in North America (24 in the USA and two in Canada). Only three studies were impact evaluations reporting quantitative outcomes [22,27,28], and none of these were randomized controlled trials (RCTs). These evaluations included sexual and reproductive health outcomes such as use of contraceptives, pregnancy prevention, and screening for sexually transmitted infections [22,27,28]. None of these papers included mental health outcomes. The remainder of the studies retrieved were those evaluating accessibility of services and clinic utilization [15-17,23,29-43], only one of which, [33] used an experimental method with a comparison group and other studies which looked at contextual factors, including operational issues [12,44,45] and cost-effectiveness [46].

**Table 1:** Characteristics of studies

**School-based healthcare (SBHC) and sexual, reproductive, and mental health outcomes**		
**First author, year, country Study design**	**Participants/Sample**	**Sexual and reproductive health (SRH), mental health (MH), and other outcomes**
Evhthier, 2011, USA Controlled before and after study	5,930 (1374 girls, 1,226 boys) students from 12 high schools in Los Angeles	Sexually experienced girls with access to SBHC had increased hormonal contraceptive use (18.1% *vs.* 12.4%), were tested for a STD (33.8% *vs.* 22.7%), received STI/pregnancy prevention care (61.4% *vs.* 53.1%) and used emergency contraception at last intercourse (3.8% *vs.* 1.8%). There were no significant differences in condom use at last intercourse. None of outcomes were significantly different between boys without access to a SBHC
Kirby, 1991, USA Controlled before and after study	Six SBHCs from different parts of the USA and four comparison schools	Although three schools reported more contraceptive use by students, overall there was no evidence SBHC reduced pregnancy rate. One school reported significantly increased use of contraceptives at last sex for boys and girls compared to the comparison school. Schools with increased usage of contraceptives and condoms emphasized pregnancy and HIV prevention throughout the school
Kisker, 1996, USA Cohort study	3,050 young people from 19 schools with SBHCs and a nationally representative sample of 859 urban youth	Reduction in age of sexual debut in students with SBHC compared to students from nationally representative sample. No difference in rate of pregnancy or contraceptive use between the students. Knowledge of effective contraceptives was 64% *vs.* 53% for those with SBHC *vs*. nationally representative sample. There was a reduction in number of students who had ever considered suicide (21% *vs.* 22%) in those with SBHC. No difference in those who had attempted suicide. However there was no difference in health status, no difference in alcohol, cigarette, and marijuana use. No difference in educational outcomes between those with SBHC compared to the comparison students
**Utilization of school-based healthcare (SBHC)**		
**First author, year, countryStudy design**	**Participants/Sample**	**Findings on utilization**
		
Adelman, 1993 USA Cross-sectional study	471 (220 boys, 251 girls) students in one Los Angeles school	44% of potential users used SBHC. Majority were girls (57%). Over 1-year period 5% had not used SBHC; 39% made 2 to 5 visits and 8% made 6 to 8 visits. 49% of all students accessed medical services; 28% MH services and 18% birth-control supplies. No differences between users and non-users in terms of demographics or school grades. Majority of non-users perceived themselves as healthy (36%). Ease of access most commonly cited reason for utilization (45%)
Allison, 2007, USA Cohort study	3,599 adolescents (790 SBHC users and 925 other users) from nine SBHCs, nine Community Clinics, and two urgent care centers in Denver	SBHC users less likely than other users to be insured (37% *vs.* 73%), more likely to have made three or more primary care visits (52% *vs.* 34%), less likely to have used emergency care (17% *vs.* 34%), more likely to have received a health maintenance visit (47% *vs.* 33%), influenza vaccine (45% *vs.* 18%), a tetanus booster (33% *vs.* 21%), and a hepatitis B vaccine (46% *vs.* 20%). Compared to traditional outpatient sites, SBHCs improve access to care for underserved adolescents
Amaral, 2011, USA Cross-sectional study	4,640 students from four schools in California	85% of sample were SBHC users and majority were girls (60%). 15% of users had accessed mental health services. Students who considered suicide in past year were 52% more likely than peers to have sought SBHC services (OR = 1.52; 95% CI:1.30, 1.78) and 112% more likely to have utilized SBHC MH services (OR = 2.12, 95% CI: 1.68 to 2.66). Users more likely to report substance abuse. Students without public medical insurance more likely to access SBHC MH services (63%; OR = 1.63, 95% CI: 1.24, 2.14). Users reported significantly lower grades than their peers
Anglin, 1996, USA Cohort study	6080 students attending three SBHCs in Denver	63% of students enrolled in the SBHC used it during the 4-year period, representing 42% of the total student population. Service users were more likely to be girls and Hispanic. The most common reasons for use were mental health problems (25%). Reproductive health advice was sought by only 11% of users. SBHC users had higher visit rates for mental health than adolescents using traditional healthcare services. SBHCs that provide a variety of medical and mental health services seem to increase utilization rates. These rates do not represent over-use, but rather appear to signify utilization patterns that occur when students have free access to needed services
Ballasone, 1991, USA Croos-sectional study	614 (313 boys, 301 girls) students in one school in Washington state	43% of all students enrolled in school used the SBHC, majority of whom were girls (53%); 58% of users accessed medical services; 20% mental health services; 6% birth control advice (clinic did not prescribe or dispense contraceptives); and 4% pregnancy test. Users were significantly more likely than non-users to exhibit high risk behaviors, for example, drug use (17% *vs.* 8%) and alcohol usage (50% *vs.* 35%). Users were consistently more likely to know where to access services, for example, birth control assistance (82% *vs.* 72%). 80% of users found services to be needed, accessible, and helpful. 70% of non-users reported not requiring services; 6% reported parental objections; and 12% were too embarrassed
Britto, 2001, USA	2,832 students in six intervention schools and 2,036 students in six matched comparison schools in Ohio	In the first year 51.2% of the intervention *vs.* 45% of comparison students did not seek care they needed. In the second year the proportion was 50.4% *vs.* 50.9%, respectively; 18.4% intervention students *vs.* 17.7% of comparison students had mental health visits in year 1 and 17.7% *vs.* 18.1% in year 2
Coyne-Beasely, 2003, USA Cross-sectional study	949 sexually experienced students (455 boys, 494 girls) in a convenience sample of seven schools with SBHC in North Carolina	Girls were more likely than boys to report needing reproductive health/STI services. 80% of girls reported they would use SBHC for reproductive/STI care, compared with 47% of boys. None-use was associated with not needing the services; confidentiality and continuing with usual healthcare providers. It is worthwhile placing reproductive and STI services in SBHCs where many adolescents have unmet health needs related to pregnancy prevention and STIs

Guo, 2008, USA Controlled before and after study	109 students with mental health problems in four schools with a SBHC and two matched comparison in Ohio	Those with a depressive disorder were more likely to use services (20% *vs.* 10.3%) compared to those without access to a SBHC. For students generally, the proportion of students accessing mental healthcare services increased (5.6% *vs.* 2.6% in urban schools and 5.9% *vs.* 0.2% in rural schools) compared to those without a SBHC. Students with mental health problems and who had a SBHC had significantly lower healthcare costs than those students without a SBHC
Harold, 1993, USA Cross-sectional study	225 (72 boys, 443 girls) students in four schools in a large Mid-Western city	More girls than boys utilized the SBHCs (92% *vs.* 8%). 59% of the sample were Caucasian; 23% African American; 10% Hispanic; 6% Asian; 4% Native American. Majority of students sought services related to pregnancy and sexual activity. 11% were pregnant at time they requested services; 58% attended for ‘family planning’ services. However, SBHC staff felt that students often used stated reasons for using clinic as means with which to start discussion about other concerns. Because students were less likely to seek services in unfamiliar settings, it is essential for SBHC staff to assess and meet the health and mental health needs of students
Ingram, 2010, UK Cross-sectional study	515 SBHC service users (72 boys, 443 women) from 16 schools in South West England	More girls than boys accessed reproductive health services (83% *vs.* 17%). Each student made an average of 2.6 visits per year. 61% said they attended SBHC because it was easily accessible. Barriers included embarrassment, cultural issues, and concerns about confidentiality. SBHCs attracted normally underserved adolescents
Jepson, 1998, USA Cohort study	2000 SBHC users from one school in New York	Mental health services represented 17% of all visits made to SBHC during a 1-year period. These students attended an average of four mental health visits per year. The majority of visits were made by girls (79%). Issues relating to pregnancy were the most common reason for seeking mental health services, whilst ongoing depression and suicidal ideation represented 22% of visits. For high-risk youth in particular, the convenience and accessibility of SBHCs can improve timely medical and mental health assistance
Juszczak, 2003, USA Cohort study	451 (176 boys, 275 girls) students from three high schools in New York	Over half (56%) of the sample used SBHC. Visits were primarily for medical (66%) and mental health (34%) services. Urgent and emergency care use was four times more likely for adolescents who had never used SBHC. SBHC can complement other health services and improve utilization of mental health services by underserved groups
Kaplan, 1998, USA Cohort study	342 students (148 boys, 194 girls) from three schools in Denver	The majority of SBHC visits were made by girls (63%). Those with access to SBHC were more than 10 times likely to make a mental health visit or substance abuse visit compared to those without access to SBHC and 98% of these visits were made to SBHC). Students with access to SBHC had 38% to 55% fewer visits per year to after-hours care (for example, emergency visits) than those without access. SBHCs are particularly effective at improving access to and treatment for mental health and substance abuse problems
Langille, 2008, Canada Cross-sectional survey	1,629 students (831 boys, 798 girls) from three schools with SBHC in Nova Scotia	More girls than boys used SBHC services (49% *vs*. 10%). Of those who used services girls were significantly more likely to use reproductive health services than boys (81% *vs*. 32%). Although sexual activity and alcohol abuse were identified among many non-users, all high-risk behaviors were significantly more likely to be exhibited by SBHC users. In this group of SBHC users, boys were seen to be more frequent binge drinkers (61% *vs*. 52%) and marijuana users (19% *vs.* 8%) compared to girls, whilst girls were seen to be more sexually active (63% *vs*. 57%), and have more frequent thoughts of suicide (18% *vs*. 13%) compared to boys. SBHC needs to find better ways to engage with boys and for reaching high-risk students
Pastore, 1998, USA Cross-sectional survey	630 students (284 boys, 347 girl) in one school with SBHC in New York	Frequent users were more likely to be girls (68% *vs.* 32%). The SBHC was used for mental health services (34%) and sexuality-related care (15%). No significant differences were found among average, frequent users, and non-users in their rates of depression, suicidal ideation and attempt, alcohol involvement, or exposure to violence. Of users and non-users with mental health problems 50% knew someone who had been murdered. Non-use was related to reporting already having a physician (60%), being healthy and not needing services (50%), and parental objection (20%). Users reported high overall satisfaction with services (92%), and felt that it was confidential (74%)
Pastore, 2004, USA Cohort study	2,090 students using SBHC in two schools in New York	In both schools girls made most visits to SBHC (72% and 63%). In both schools visits made were for mental health issues (11% and 19%) and reproductive health issues (12% and 20%). SBHC serves students’ reproductive and mental healthcare needs and they should provide comprehensive medical and mental health services to improve access for adolescents
Soleimanpur, 2010, USA Cross-sectional survey Focus groups	7410 students using 12 SBHCs in California	SBHCs were the most commonly reported source of medical (30%), family planning (63%), and counseling (31%). Significant improvements were reported in mental health outcomes and reproductive health. Students liked SBHCs because of perceived confidentiality of services, because they were free and convenient and because they found the staff friendly. SBHCs increased access to care and improved mental health, resilience, and contraceptive use
		
		
Szumilas, 2010, Canada Secondary analysis of cross-sectional survey	1, 629 students (831 boys, 798 girls) from three schools with SBHC in Nova Scotia	More girls than boys used the SBHC for mental health support in the preceding school year (20.4% *vs.* 5.3%) with girls most often asking for relationship support and boys for support with substance use. Students who used SBHC significantly more likely to report lower school performance, more sexual health risk-taking, suicidal behavior, and risk for depression. Boys reported confidentiality concerns. There was substantial need for mental health support and significant unmet need, particularly for boys
Walter, 1996, USA Cross-sectional survey	3,738 (1,992 boys, 1,746 girls) students in four schools with SBHC in New York	Just over one-third (36%) of the study sample had utilized SBHC services during the academic year.Except grade differences, no other demographic differences were observed between users and non-users. Higher number of users compared to non-users reported sexual intercourse (22% *vs.* 18%), failure to use birth control (22% *vs.* 13%), suicide intentions or attempts (16% *vs*. 12%). SBHC can attract and provide a range of primary and preventative health services for underserved adolescents who may be most in need of such services
Weist, 1995, USA Cross-sectional survey	164 (77 boys, 87 girls) students in one inner city school in Baltimore	34% of the sample were clinic users, of whom 52% were girls. Frequent users were significantly more likely to be girls (12/14 students). In general frequent users were more depressed and anxious than other groups. No significant differences were observed between users and non-users on psychosocial measures
Wolk, 1993, USA Cohort study	1,413 students in one Denver school	Girls were significantly more likely to be frequent users than average users. Frequent users were significantly more likely to be diagnosed with mental health conditions (23%) compared to average users (3.7%); 61% of all SBHC visits were for mental health purposes. The high prevalence of risky behaviors by users of SBHC emphasized the importance of SBHC within high schools
**Contextual issues in the provision of school-based healthcare**		
**First author, year, country sampleStudy design**		**Contextual issues**
Billy, 2000, USA Secondary analysis of cohort study	104 high schools (91 public, 13 private)	Schools with students experiencing more health risks were more likely to provide school-based health services. State policies were important and community provision of health services influenced provision in schools. More affluent communities were more likely to provide SBHC. Contextual factors appear to create a demand for services
Santelli, 2003, USA Cross-sectional survey	551 SBHCs in 313 schools in the US	SBHC was more common in urban (55%) and rural (33%) than suburban (12%) areas. Most (76%) were open full-time and 48% were open during school holidays. Counseling, pregnancy testing, STD/HIV services were often provided on site (range 55% to 82%), whilst on-site availability of contraception ranged from 3% to 28% and was often provided by referral externally. Most schools (76%) reported prohibitions about providing contraceptive services on site. More established SBHCs were more likely to allow independent adolescent access without parental permission
Peak, 1996, USA Cross-sectional survey	180 school health services (109 SBHCs and 16 school-linked)	Established centers in urban and suburban areas provided the broadest range of services. Thirty-three per cent made at least one contraceptive method available. Restrictions on these services came mainly from school district policy. Although such services offer a promising solution to delivering sexual and reproductive health care external and internal policies restrict their availability and scope

## Discussion

### School-based health clinics and adolescent sexual, reproductive, and mental health outcomes

Despite the relatively established precedent of SBHC in North America [25] there is surprisingly little robust scientific evidence of its effectiveness in terms of sexual and reproductive or mental health outcomes. There are no known randomized controlled trials and the results of studies that have used a comparison group have been mixed. Nevertheless, some studies have shown that students received more focused preventative healthcare. For example, Ethier and colleagues [27] found that girls at schools which provided school-based health centers had an increased odds of reporting having received pregnancy or disease prevention care (adjusted odds ratio, AOR=1.68, 95% CI, 1.16 to 1.80), having used hormonal contraceptives at last sex (AOR= 1.68, 95% CI, 1.24, 2.28) and were more likely to have been screened for sexually transmitted diseases (STDs) (AOR=1.85, 95% CI, 1.43, 2.40). Also, female students at schools with SBHC were more likely to have used emergency contraception at last sex (AOR=2.1, 95% CI 1.08, 4.22). However access to SBHC did not influence receipt of reproductive healthcare for boys. Kirby [22] reported mixed findings with regard to the role of SBHC in sexual and reproductive health outcomes in six intervention schools compared to four matched comparison schools. Overall there was no evidence that SBHC reduced pregnancy rate. However, the schools all offered slightly different services. For example only three of the six ‘intervention’ schools actually provided contraceptives on-site and only one of these schools reported a significantly increased use of contraceptives at last sex by young people in that school *versus* the comparison school (boys, 78% *vs.* 61%, *P* <0.001 and girls, 75% *vs*. 60%, *P* <0.001). Combining SBHC with an in-school educational program that focused on HIV in a community with high prevalence encouraged a sharp rise in reported condom use in another school (boys 61% *vs*. 41%, *P* <0.001). The authors suggest that focusing on issues which are priorities for specific school communities may have merit rather than a ‘blanket’ approach to provision. However this does make comparisons of school programs and measures of overall effectiveness of SBHC difficult. Kisker and colleagues’ study [28] compared outcomes of students attending 19 schools in the US with school-based health centers sponsored by a particular funding agency to a nationally representative sample of students. They reported mixed and inconsistent findings. For example they suggest that SBHC may have reduced initiation of sexual activity (67% *vs.* 70%, *P*=0.05) but that SBHC students were less likely to use contraceptives at last sex than the nationally representative sample (75% *vs.* 80%, *P*=0.05) and there were no differences in the rate of pregnancy (25% *vs*. 25%). They also reported that SBHC had reduced the number of students who had ever considered suicide (21% *vs*. 22%, *P*=0.01) yet there was no difference in those who had attempted suicide when compared with the national sample. One explanation for these inconsistent findings may be that there were quite substantial methodological issues with the study in that the comparison students may have had access to SBHC and therefore did not act as a true comparison group.

### Access to and utilization of services

Much of the evidence about SBHC has come from descriptive studies which have examined access to and use of services and the vast majority of the papers retrieved were related to access and clinic utilization. It appears that overwhelmingly girls tend to use services more than boys [16,17,23,24,30-32,34,38-40,42,43,47], and that students who reported experiencing the greatest level of mental health difficulties such as those who had considered suicide, had sleep disturbance and depression were more likely to use SBHC than those without such difficulties [17,36,47]. Some studies found that the more frequent users of SBHCs reported higher levels of mental health need than their peers [17,30,31,36,42] although one of the studies did not support this [40]. Jepson and colleagues found that the primary reason for using mental health services were for pregnancy and sexuality issues, depression or conflict, and violence [36]. Users of SBHC were also often described as exhibiting more high risk behaviors including unprotected sexual intercourse, substance usage, and suicidal behavior than non-users of services [12,15,17,29,38,41,43,47]. Adolescents exposed to SBHC received more mental health services compared with those in schools without SBHC and there appeared to be a cost efficiency saving comparing them to those not exposed [35,46]. It appears therefore, that SBHC can reach adolescents with the greatest level of need.

SBHC has been cited as being able to enhance adolescents’ access to care for medical, mental health, and substance abuse problems [16] but it appears that even then a significant proportion of students remain underserved particularly for mental health services such that many students despite having access to SBHC still reported not seeking the care that they needed [31,33,48]. School-based health services, therefore, do not always fully substitute as the primary source of healthcare and students still report having emergency department visits, although sometimes less often than those without access to SBHC [32]. It is therefore important to investigate methods to implement SBHC so that they maximize accessibility and capacity to provide such services.

### Reasons for use and non-use of SBHC

A number of studies explored the reason for use and non-use of school-based healthcare. The most commonly cited reason for non-use was related to students’ perception of being healthy and therefore not requiring school-based health services; already having a physician and to a lesser degree, concerns about confidentiality and parental objections [15,31,34,40]. Ease of access was the most frequently mentioned reason for use [31,34]. Ninety-one per cent of students felt that they had got the service they wanted [23] and 92% were very or somewhat satisfied with the services [40]. Mental health services and reproductive health services and medical services were the most commonly reported reasons for using SBHC [37,40,47] and a high proportion of sexually experienced students reported they would use SBHC for reproductive health and STI services where available [34]. Moreover, these services appealed most to female students from lower socioeconomic backgrounds and girls who inconsistently used contraception [34], highlighting the potential of SBHC services to reach adolescents considered to be ‘high risk’, who would benefit the most from receiving such preventative and early interventions.

Whilst adults planning SBHC may feel that pregnancy prevention is the top priority it is also important to ensure that such clinics meet the needs of young people which often include the need to discuss physical body changes, relationships, family issues, and psychological wellbeing [47]. A study conducted in the UK that analyzed patterns of use, reasons for attendance, and views on services in 16 high schools found that the service attracted normally underserved students including boys, those less academically able and those engaging in sex at younger ages [23]. A wide range of sexual and reproductive health services were taken up. However barriers to services included embarrassment, cultural issues, and fears about confidentiality. Alternative ways need to be found to engage with non-users, particularly boys [47]. Interestingly, among all respondents in the UK study, a significant percentage reported most commonly seeking help from friends and family members, highlighting the potential value of peer-support and counselor training programs [23].

### Contextual influences

Billy and colleagues [44] looked at contextual influences on the provision of SBHC and found, as one might expect, that those States in the US that had policies around school-based provision and had students enrolled who had health-related risk, were more likely to provide such services. Availability of accessible healthcare services within the local community tended to impede provision of school-based health services and in general more affluent communities tended to provide SBHC more than less affluent communities. The authors stated that highlighting the health concerns of students in specific communities can stimulate the provision of services needed.

The importance of well-trained staff who are able to communicate with adolescents, referral systems and adequate follow-on care, and collaboration between health service staff and schools can improve service delivery and effectiveness [8].

The importance of planning for the setting up of SBHC cannot be overemphasized [49]. If accessible and sensitive services are already available locally such a service may not be needed. It is also important to explore any sources of both resistance and support in the community. Often SBHC has been seen as ‘contraceptive clinics’ that will encourage young people to become prematurely sexually active, often resulting in school district policy restrictions [29]. However, such concerns are not supported by the evidence [22]. Nevertheless, planners must be ready to listen and ensure that comprehensive services are offered. This may also help in making visiting SBHC less stigmatizing for students. Schools and healthcare staff may also resist the setting up of SBHC due to the blurring of boundaries between professionals, increased responsibility, and workload. It is recommended that a representational advisory board is set up in the planning stages for each school so that the SBHC model adopted reflects the needs of the community in which it is based, and does not rely on a ‘one-size-fits-all’ approach. Another important consideration relates to the integration of SBHC services into existing systems of care. The location of SBHC in deprived communities may result in these centers becoming key primary care providers so effective coordination of referral systems to other sources of care is essential as it developing strong partnerships with communities [12,32,45].

The source of funding for SBHC is an issue that needs to be addressed because of the long-term needs of adolescents in schools. In most of the reported studies funding came centrally from government. Evaluation of cost-benefit of SBHC was undertaken by Guo and colleagues [46] using a quasi-experimental repeated measures design and using data from medical aid claims and parental reports. They found that SBHC was cost beneficial in terms of the medical aid system and argued that it is also cost beneficial to society in reducing health disparity gaps.

### Limitations

Our search was limited to published English language peer-reviewed studies; there may be grey literature available which provides more evidence of effectiveness of school-based health centers for sexual, reproductive, and mental health of adolescents. However this was very difficult to identify, systematically. The authors also did not have access to the CINAHL database and there may have been nurse-led studies which have been missed. Most of the studies identified were descriptive. Only five studies employed a comparison group [22,27,28,33,35] but none were randomized controlled trials, hence comparisons between schools with and without SBHC may be biased. The review was therefore severely limited by the quality of the available studies. No formal risk of bias assessments were carried out which is also a potential limitation of the review. Most of the studies used self-reported measures of healthcare usage and health status or else used retrospective reviews of health records. Some of the studies [17,28,31,34,37,44] mentioned other services young people utilized outside of school such as STI treatment, maternity, general practitioner, pharmacy, clergy, or alternative healing services, for example, that may have impacted on utilization as a whole and health outcomes in particular but none analyzed this in any depth. Also, reports of utilization were from students attending school. Their health needs and patterns of healthcare utilization are probably not representative of those who have dropped out who are more likely to have a higher burden of disease [18]. It is feasible that users of SBHC are different from non-users. In terms of analysis of studies, few authors employed rigorous statistical techniques to control for confounding factors such as age, gender, ethnicity, and health status.

Finally and most importantly, clearer definitions are needed of what SBHC actually is, as it is different in different contexts and countries. Currently the range of what constitutes SBHC makes comparisons across a range of settings difficult. For example, services could range from comprehensive primary care services provided Monday to Friday during school hours by a doctor, nurse, counselor/psychologist, social worker, and dentist providing screening, diagnosis, lab tests, treatment for minor illnesses and injuries, immunizations, gynecological examinations, STI treatment, and contraceptive advice and provision [22,28], or a nurse and youth worker service operating for one lunchtime a week during term time [23].

## Conclusions

It appears that SBHC can provide services for normally underserved young people particularly for mental health services. However, more high-quality research is urgently needed into the effectiveness of SBHC on sexual reproductive and mental health outcomes and especially on what encourages young people to use services and what prevents others, particularly boys, from not using services. Careful planning and collaboration with young people, parents, and local communities is clearly needed. School-based health services may not be the main source of healthcare due to their limited coverage of schools, services offered or opening times (that is, closed at weekends and during holidays) and health policy-makers need to ensure that there is a seamless provision between usual healthcare delivery networks. School-based health services should ideally form a complementary service and should not replace other community health provision [37].

Currently the evidence is both severely limited and equivocal on the effectiveness of SBHC on adolescent sexual, reproductive, and mental health. If further studies can begin to define what the minimum standards for comprehensive SBHC services are and can ascertain ‘what works’ using appropriate and rigorous research methodologies it could provide promise as an intervention for addressing key adolescent public health issues. But until that time, although intuitively and anecdotally it may seem to make good sense we do not have the evidence to be confident to make claims as to its effectiveness.

## Competing interests

AMJ was previously funded by the South African MRC and the PREPARE PROJECT and is currently funded by the University of York. CC was funded by Brown University. MM, JK PDK and CM are funded by the PREPARE.

## Authors’ contributions

AMJ designed and led the study, reviewed all included studies, and wrote the paper. CC completed the search, reviewed all studies, and co-wrote the paper. MM reviewed all studies and co-wrote the paper. CM, JK, and PDK reviewed a proportion of included studies and gave feedback on the manuscript. All authors have read and approved the final manuscript.
